# Enhancing Volumetric Hydrogen Storage Capacity through Bimodal Packing of MOF Particles

**DOI:** 10.1021/acsomega.6c02165

**Published:** 2026-05-05

**Authors:** Wan-Tae Kim, Dae Won Kim, Dong Yun Shin, Hong-Eun An, Albert S. Lee, Jung-Hoon Lee, Chang Seop Hong, Sohee Jeong

**Affiliations:** † Extreme Materials Research Center, 58975 Korea Institute of Science and Technology (KIST), Seoul 02792, South Korea; ‡ Department of Chemistry, Korea University, Seoul 02841, South Korea; § Computational Science Research Center, Korea Institute of Science and Technology (KIST), Seoul 02792, South Korea; ∥ Department of Materials Science and Engineering, Korea University, Seoul 02841, South Korea; ⊥ Convergence Research Center for Solutions to Electromagnetic Interference in Future-Mobility, Korea Institute of Science and Technology (KIST), Seoul 02792, South Korea; # KU-KIST Graduate School of Converging Science and Technology, Korea University, Seoul 02841, South Korea

## Abstract

Packing density critically influences the practical hydrogen storage capacity of metal–organic frameworks (MOFs), yet it is often overlooked in volumetric performance evaluations. In this study, we demonstrate that bimodal particle packing provides an effective route to enhance system-level volumetric hydrogen storage by reducing interparticle voids. A V_3_(PET) MOF was synthesized in two distinct sizes (∼9 μm and ∼300 nm) to construct a bimodal particle system. Discrete element method (DEM) simulations were used to establish particle-packing design rules and to identify the optimal mixing composition for maximizing packing density. These simulation-guided predictions were then experimentally validated through tapping density measurements and high-pressure H_2_ adsorption isotherms. The optimized bimodal mixture achieved a packing fraction of 0.56, compared to 0.42 for unimodal packing, leading to a 33–38% increase in volumetric excess hydrogen uptake at 77 K. Moreover, the bimodal system exhibited an enhanced working capacity of up to 37.7 g/L under pressure–temperature swing adsorption (PTSA) condition (160 K 5 bar to 77 K 100 bar). These results demonstrate particle-level packing engineering as a broadly applicable and experimentally accessible strategy for bridging intrinsic MOF properties with realistic, system-level hydrogen storage performance.

## Graphical abstract

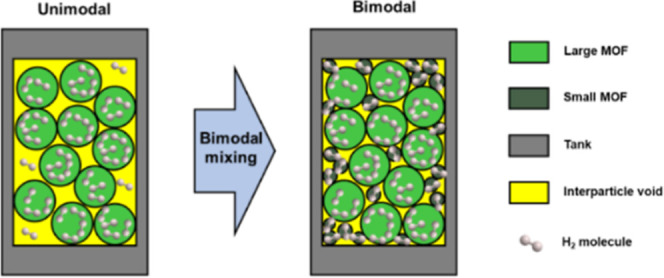

## 1 Introduction

The transition to a hydrogen economy demands effective and safe hydrogen storage methods. Solid-state storage using metal–organic frameworks (MOFs) offer a promising alternative to compressed and liquid hydrogen, as it enables storage under more moderate pressure and temperature conditions while maintaining high adsorption–desorption reversibility and kinetics.^1,2^ Owing to their exceptionally high specific surface areas and tunable porosity, MOFs have demonstrated excellent gravimetric hydrogen storage capacities.^3^ However, despite their superior gravimetric performance, MOFs often suffer from limited volumetric hydrogen uptake, primarily due to two factors. First, the inherent increase in porosity, although favorable for gravimetric storage, typically results in a reduced crystal density.^3–5^ MOFs with excessively large pores gain less adsorptive advantage relative to the associated increase in volume, creating a fundamental trade-off between gravimetric and volumetric capacities. Second, in practical applications, packing inefficiencies between MOF particles introduce interparticle voids, further exacerbating the loss of volumetric storage capacity.

While volumetric hydrogen uptake is frequently calculated based on theoretical crystal density—assuming ideal, void-free packing—this idealized condition is rarely achieved in practice (Scheme 1A).^2,4^ In reality, the actual packing density is significantly lower, resulting in substantial interparticle voids and a reduction in effective volumetric uptake (Scheme 1B).^6,7^ These losses point to the critical need for strategies that can enhance packing density while preserving the intrinsic properties of the material. Some attempts have been made, such as densifying MOF powders into monoliths or pellets; however, these do not reflect the packed shape in actual storage tanks.^8,9^ In other fields such as powder metallurgy and concrete engineering, bimodal particle size mixing—blending large and small particles—has proven effective for enhancing packing efficiency by minimizing interstitial void space.^10^ Although this concept has occasionally been considered for hydrogen storage materials,^6^ comprehensive experimental demonstrations in MOFs remain scarce. Notably, previous efforts using size-mixing MOF-5 powders resulted in only marginal improvements, highlighting the need for a deeper understanding and experimental validation of bimodal packing effects in practical MOF systems.

**Scheme 1:**
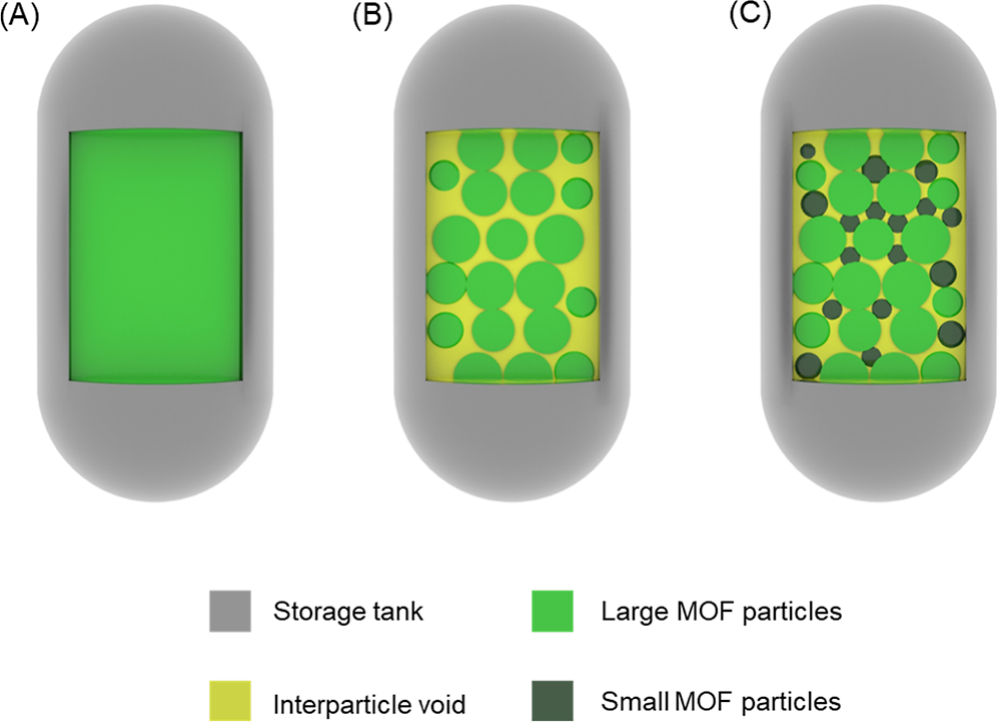
Conceptual Schematic Illustrating the Difference Between Theoretical and Practical Packing Scenarios for Hydrogen Storage Materials; (A) Idealized Case Assuming Crystal Density; (B) Realistic Packing of Unimodal Particles; (C) Improved Packing Achieved via Bimodal Particle Packing

Motivated by these challenges, we sought to address the volumetric limitations of MOFs by applying a bimodal packing strategy. We selected V_3_(PET) (H_6_PET = 4,4′,4″,4‴,4‴′,4‴″-(9,10-dihydro-9,10-[1,2]­benzenoanthracene-2,3,6,7,14,15-hexayl)­hexabenzoic acid), a recently developed MOF that exhibits a remarkable hydrogen storage capacity of 8.4 wt % and 46.7 g/L at 77 K and 100 bar.^11,12^ Its high gravimetric and volumetric performances, combined with excellent reversibility, make it an ideal candidate to investigate the effect of bimodal particle mixing on packing and storage performance.

In this study, we systematically investigated how bimodal particle packing influences volumetric hydrogen storage performance (Scheme 1C). We synthesized V_3_(PET) MOF particles in two distinct size regimes (∼9 μm and ∼300 nm), hereafter denoted as V_3_(PET)_large and V_3_(PET)_small, respectively. To optimize the bimodal packing, we employed discrete element model (DEM) simulations to predict optimal size ratios and mixing compositions. By experimentally validating tapping density and high-pressure hydrogen adsorption performance, we demonstrated that bimodal packing substantially enhances volumetric excess uptake and working capacity without compromising material integrity.

## 2 Materials and Methods

### 2.1 Synthesis of V_3_(PET)_large

V_3_(PET)_large was synthesized by a previously reported method with minor modifications.^11^ VCl_3_(tetrahydrofuran)_3_ (695 mg) and 4,4′,4″,4‴,4‴′,4‴″-(9,10-dihydro-9,10-[1,2]­benzenoanthracene-2,3,6,7,14,15-hexayl)­hexabenzoic acid (H_6_PET, 150 mg) were mixed in 25.0 mL of *N*,*N*-dimethylformamide (DMF), 25.0 mL of acetonitrile, and 2.5 mL of acetic acid in a 250.0 mL glass bottle, and then the mixture was sonicated for 30 min. The mixture was heated to 150 °C for 48 h. The precipitated crystalline powder sample was settled down, and the mother liquor was removed. The solid product was washed 3 times with 30.0 mL of DMF and immersed in 30.0 mL of DMF overnight. Then, DMF was removed, washed 6 times with 30 mL of acetone, and immersed in 30.0 mL of acetone for 2 days. The acetone was decanted, and the samples were activated under vacuum at 150 °C for 12 h to yield 136 mg (75%) of V_3_(PET)_large as a green powder.

### 2.2 Synthesis of V_3_(PET)_small

V­(acetylacetonate)_3_ (774 mg) and H_6_PET (180 mg) were mixed in 10.5 mL of DMF, 10.5 mL of acetonitrile, and 2.0 mL of hydrochloric acid (37%) in a 50 mL glass vial, and then the mixture was sonicated for 30 min. Then, the sonicated solution was transferred into a 50 mL round-bottom flask equipped with a magnetic stirrer bar. A condenser was attached to the flask, and N_2_ was filled using a rubber balloon. The flask was heated to 135 °C with stirring at 600 rpm for 72 h. The precipitated V_3_(PET)_small powder was centrifuged to separate from the mother liquor. The product was washed 3 times with 30 mL of DMF and immersed in 30 mL of DMF overnight. It was centrifuged and washed 6 times with 30 mL of acetone and immersed in 30 mL of acetone for 2 days. The acetone was decanted, and the samples were activated under vacuum at 150 °C for 12 h to yield 182 mg (82%) of V_3_(PET)_small as a light green powder.

### 2.3 Characterization of V_3_(PET)

Field emission scanning electron microscope (FE-SEM, Inspect F, FEI Company), powder X-ray diffraction (PXRD, D8 Advance, Bruker AXS Inc.) were used for materials characterization. N_2_ adsorption–desorption isotherms at 77 K were collected on a Micromeritics Tristar II Plus 3030 instrument after activating the samples at 150 °C under vacuum as described above. Brunauer–Emmett–Teller (BET) surface areas were calculated using the Rouquerol criteria.^13^ The pore size distribution and pore volume (*V*_p_) were obtained using the nonlocal density functional theory (NLDFT) slit-pore kernel implemented in Micromeritics MicroActive.

### 2.4 Tapping Density Measurement

All samples used for tap density measurements were fully activated under vacuum at 150 °C for 12 h. After activation, the samples were handled entirely inside an Ar-filled glovebox, and the syringe tapping measurement was also performed entirely inside the glovebox to prevent moisture readsorption. The tapping density of the V_3_(PET) samples was measured using a 1 mL glass syringe. For each sample, specific weight ratios of V_3_(PET)_small to V_3_(PET)_large were prepared in the following proportions: 10:0, 8:2, 6:4, 4:6, 2:7, and 0:10. These mixtures were gradually added to the syringe, which was tapped against a solid surface until no further decrease in volume was observed, indicating that the maximum packing density had been achieved. The final volume occupied by the sample was recorded, and the tapping density was calculated as the mass of the sample divided by the final tapped volume.

### 2.5 High-Pressure H_2_ Isotherm Measurement

To assess the hydrogen storage capacity, we measured high-pressure H_2_ isotherms for V_3_(PET)_large, V_3_(PET)_small, and the optimized bimodal mixture (V_3_(PET)_bimodal) with the maximum packing density at 77, 160, and 298 K. Each sample was activated under vacuum at 150 °C for 12 h using a Micromeritics VacPrep 061 degassing instrument to remove any residual solvents and moisture. After activation, the degassed sample was gradually loaded into the 1.5 mL customized sample cell by hand tapping the cell until the maximum sample loading was achieved in a glovebox. The weight of the sample cell was measured before and after loading the sample inside the glovebox to determine the precise sample mass. The sample cell was then sealed with a Swagelok SS-8-VCR-2-GR-5 M stainless steel gasket, transferred out of the glovebox, and mounted onto the automated controlled Sieverts apparatus (BEL-HP, BEL.JAPAN.INC).

Then, the sample was regenerated at 150 °C for 6 h under vacuum. Subsequently, the skeletal volume of the sample at 25 °C was measured by dosing helium up to 1 bar using the BEL-HP instrument,^14,15^ primarily for accurate dead volume correction. Both the skeletal volume of the sample and the dead volume of the empty cell were determined by dosing helium at 25 °C a total of 60 times, with the average values used for calculations. Following this, excess hydrogen uptake was measured up to 100 bar at 77, 160, and 298 K using a liquid nitrogen level controller, a customized cooling block with a cryogenic temperature controller (model 335, Lake Shore Cryotronics, INC.), and an ethylene glycol-water bath, respectively.^16^

To investigate how bimodal packing addresses volumetric storage, we calculated the system-level volumetric hydrogen uptake by incorporating the experimentally measured packing densities for each sample configuration. System-level volumetric excess uptakes were estimated from the experimentally measured gravimetric excess uptake using the following equation

$$n_{vol}\;\left(g/L\right)=n_{grav}\;\left(g/kg\right)\cdot \rho_{packing}\;\left(g/{cm}^{3}=kg/L\right)$$

where *n*_vol_ is excess system-level volumetric H_2_ uptake per packing volume, *n*_grav_ is gravimetric excess H_2_ uptake, ρ_packing_ is packing density of MOFs.

Furthermore, total uptake is a more relevant metric than excess uptake, because it includes not only the adsorbed hydrogen (excess uptake) but also the non-adsorbed hydrogen that would occupy the pore and interparticle spaces in the absence of adsorption forces. Accordingly, the total gravimetric H_2_ uptake was estimated from the experimentally measured gravimetric excess uptake using the following equation^11^

$$N_{grav}\;\left(g/kg\right)=n_{grav}\;\left(g/kg\right)+1000\cdot \rho_{gas}\;\left(g/{cm}^{3}\right)\cdot V_{p}\;\left({cm}^{3}/g\right)$$

where *N*_grav_ is total gravimetric H_2_ uptake, *n*_grav_ is gravimetric excess H_2_ uptake, ρ_gas_ is the density of H_2_ gas at the given temperature and pressure, and *V*_p_ is the intrinsic pore volume of the MOF framework (cm^3^/g), excluding interparticle space.

And the total system-level volumetric H_2_ uptake was estimated using the following equation^9^

$$N_{vol}\;\left(g/L\right)=n_{grav}\;\left(g/kg\right)\cdot \rho_{packing}\;\left(g/{cm}^{3}\right)+1000\cdot \rho_{gas}\;\left(g/{cm}^{3}\right)\cdot \left(1-\rho_{packing}\;\left(g/{cm}^{3}\right)/\rho_{skeletal}\;\left(g/{cm}^{3}\right)\right)$$

where *N*_vol_ is system-level total volumetric H_2_ uptake per packing volume, *n*_grav_ is gravimetric excess H_2_ uptake, ρ_packing_ is packing density of MOFs, ρ_gas_ is the density of H_2_ gas at specific temperature and pressure, and ρ_skeletal_ is experimentally measured density of skeleton of MOF (except pore). In contrast, for the calculation of material-level total volumetric uptake, a fixed value of crystal density (0.507 g/cm^3^) was used for all samples instead of the experimentally measured packing density, in order to directly compare the effect of intrinsic porosity independent of packing configuration.

### 2.6 Packing Density Simulation

To investigate the effect of particle size disparity in binary systems on packing density, we used Particula (version 1.3),^17^ an open-source software for simulating granular media based on a discrete element model (DEM). The packing simulations were performed in a cylindrical container with both diameter and height set to 10 (arbitrary units). In all simulations, the diameter of the large particles was fixed at 3 (arbitrary units), and in binary systems the small particle diameters were set to 1.5, 0.75, 0.50, 0.37, and 0.30 (a.u.), corresponding to size ratios of 2:1, 4:1, 6:1, 8:1, and 10:1. The simulations employed a spherical particle model that accounts for particle–particle and particle–wall interactions, including frictional contacts and inelastic collisions, using the default interaction parameters provided in Particula. Specifically, the static and dynamic friction coefficients were both set to 0.6, and the particle bounciness was set to 0.1 to represent limited rebound during contact. Particles were sequentially deposited at random horizontal positions and allowed to settle under gravity. For each size ratio, the initial volume fraction of large particles, defined as *V*_large_/(*V*_large_ + *V*_small_), was varied from 0.0 to 0.9 in uniform increments of 0.1. Here, *V*_large_ and *V*_small_ are the volumes of the large and small particles, respectively. This fraction refers to the total volume of particles initially loaded into the cylindrical container. Each combination of size ratio and volume fraction was simulated as an independent packing run, with large and small particles deposited simultaneously according to the prescribed volumetric fraction rather than sequentially. To enhance packing efficiency and prevent local jamming, gentle shaking was applied laterally until no further compaction was observed. The packing fraction was determined as the ratio of the total particle volume to the container volume, and in binary systems the final volume fractions of each particle type were extracted from the equilibrated configuration.

## 3 Results and Discussion

### 3.1 Simulation-Guided Optimization of Bimodal Packing

Packing density plays a critical role in determining the volumetric hydrogen storage capacity of metal–organic frameworks. Interparticle voids, which are inevitable when MOF powders are packed into practical containers, significantly diminish achievable volumetric performance. To address this limitation, bimodal particle mixing—blending large and small particles—has been widely applied in fields such as concrete design and powder metallurgy to enhance packing efficiency.^10^ However, its application in MOF-based hydrogen storage remains largely unexplored.

To systematically investigate how particle size ratio and composition influence packing behavior, we conducted DEM simulations using Particula, a gravity-based packing simulator, in which both the diameter ratio of large to small particles and the initial volume fractions of the two components were systematically varied to identify optimal packing conditions (Figures 1, S1–S5, Tables S1–S5). Specifically, the diameter ratio of large to small particles was varied from 2:1 to 10:1, and in the bimodal systems, the initial volume fraction of large particles (*V*_large_/(*V*_large_ + *V*_small_)), as defined in the Materials and Methods section, was systematically adjusted from 0 to 0.9 to examine how the relative fraction of small particles affects the resulting packing fraction and composition.

**Figure 1:**
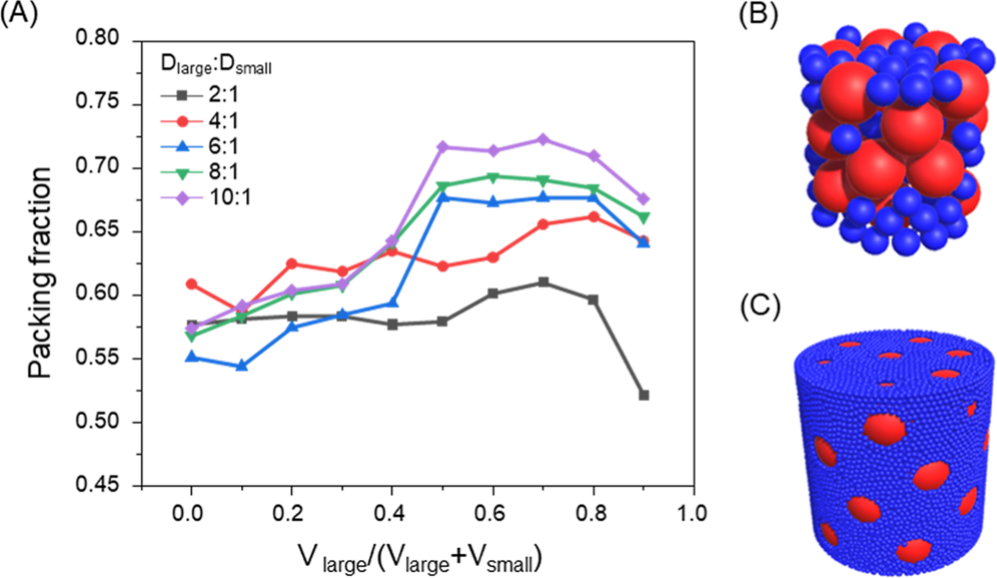
Effect of particle size ratio on packing behavior obtained from Particula simulations. (A) Simulated packing fraction as a function of particle size ratio and initial volume fraction of large particles (*V*_large_/(*V*_large_ + *V*_small_)). Representative DEM snapshots of bimodal packings at an initial volume fraction of large particle of 0.7 with diameter ratios of 1:2 (B) and 1:10 (C), illustrating the spatial distributions of large (red) and small (blue) particles.

When only small particles were present (i.e., the initial volume fraction of large particles was 0), corresponding to a unimodal system, our simulated packing fraction was in the range of 0.55–0.61, consistent with literature values for random packing of monodisperse spheres (Figure 1A).^18^ In contrast, bimodal particle systems exhibited a dependence of the packing fraction on both the particle size ratio and the large-particle fraction. As the *V*_large_/(*V*_large_ + *V*_small_) increased, the packing fraction increased across all particle size ratios. At intermediate large-particle fractions (approximately 0.5–0.8), large particles formed a packing framework, while small particles occupied the interstitial spaces between adjacent large particles, corresponding to the highest packing fractions observed. At higher large-particle fraction, the reduced number of small particles was insufficient to effectively fill the interparticle spaces between large particles, resulting in a decrease in packing fraction.

At *V*_large_/(*V*_large_ + *V*_small_) of 0.5–0.8, corresponding to the maximum packing fraction, increasing the diameter ratio of large to small particles led to a systematic increase in the packing fraction (Figure 1A). The maximum packing fractions were 0.61, 0.66, 0.68, 0.69, and 0.72 for diameter ratios of 2:1, 4:1, 6:1, 8:1, and 10:1, respectively. This trend indicates that increasing particle size ratio enhances packing efficiency by enabling more effective utilization of interstitial spaces in bimodal systems. Representative DEM snapshots further illustrate this effect. For the 2:1 system (Figure 1B) at a large-particle fraction of 0.7, small particles partially occupy the interstitial voids between large particles, but a substantial fraction of void space remains unfilled due to the limited size contrast. In contrast, the 10:1 system (Figure 1C) shows that small particles preferentially fill the interconnected void network formed by the large-particle framework, effectively reducing the overall void volume and resulting in a markedly higher packing efficiency. To further understand this packing behavior, the relationship between the initial and final volume fractions of large particles was analyzed (Figure S6), showing that when the initial volume fraction of large particles exceeds ∼0.5–0.6, the final composition becomes relatively insensitive to the initial ratio, as a large-particle framework is established and subsequent packing is governed by small particles filling interstitial voids, corresponding to maximum packing efficiency.

Taken together, these results establish two key design principles for bimodal packing. First, increasing the particle size ratio enhances packing fraction by promoting more efficient filling of interstitial voids, although the improvement becomes progressively smaller at higher size ratios. Second, regardless of particle size ratio, the maximum packing fraction is consistently achieved at an intermediate volume fraction of large particles, approximately 0.5–0.8, where large particles form a load-bearing framework and small particles effectively occupy the interparticle voids. These trends suggest behavior beyond the simulated range based on fitting analysis, which indicates a diminishing increase in packing fraction and a tendency toward saturation at higher particle size ratios (Figure S7 and Table S6).

Idealized conditions assumed in the DEM simulations may deviate from real-world experimental systems. Nevertheless, they provide useful insight into phenomenological trends in packing behavior, while being most appropriately used in conjunction with experimental results in Section 3.3 to understand overall trends. In this context, the DEM simulations are intended to provide qualitative design guidance rather than quantitative prediction of packing density or volumetric performance.

### 3.2 Synthesis and Characterization of V_3_(PET) Particles

To experimentally validate the simulation-predicted benefits of bimodal packing, we selected V_3_(PET) as the model MOF system due to its previously demonstrated high volumetric hydrogen uptake and excellent working capacity under cryogenic conditions.^11^ This material offers high intrinsic porosity and robust structural stability, making it an ideal candidate for investigating particle morphology engineering strategies.

Although the simulations indicate that increasing particle size ratio enhances packing fraction, translating these design principles into experimentally realizable MOF particles is nontrivial. Achieving precise particle size control remains technically challenging,^19^ largely because MOF crystallization is governed by reversible coordination interactions between metal clusters and organic linkers that regulate nucleation and growth.^20^ In systems employing large, rigid, and multidentate ligands such as H_6_PET, fine-tuning of reaction parameters (e.g., modulator concentration or temperature) to achieve specific intermediate sizes often risks incomplete framework formation or loss of crystallinity.^21^ Therefore, rather than pursuing incremental size tuning which may compromise structural integrity, we adopted a pragmatic bimodal synthesis strategy in which two well-separated particle populations were deliberately produced: V_3_(PET)_large (∼9 μm) and V_3_(PET)_small (∼300 nm). This corresponds to a particle size ratio of approximately 30:1, representing an experimentally accessible design that enables robust and reproducible realization of bimodal packing. Because the simulations indicate that packing enhancement increases with particle size ratio but becomes progressively less sensitive beyond moderate size disparities, the large size contrast employed here (∼30:1) places the system well within the regime where bimodal packing effects are effectively realized. This interpretation is further supported by fitting-based extrapolation of the simulation results, which shows a diminishing increase in packing fraction and a tendency toward saturation at higher size ratios (Figure S7 and Table S6).

First, V_3_(PET)_large was synthesized by solvothermal reaction of H_6_PET ligand and VCl_3_(THF)_3_, following previously reported methods.^11^ In contrast, V_3_(PET)_small was synthesized by reacting H_6_PET with V­(acetylacetonate)_3_ under vigorous stirring and reflux conditions, promoting faster nucleation and smaller particle formation. Both products underwent solvent exchange with DMF and acetone, followed by vacuum activation at 150 °C for 12 h to remove residual guests and ensure full porosity development. Detailed synthetic protocols are described in the Materials and Methods section.

Field-emission scanning electron microscopy (FE-SEM) images (Figure 2A,B) revealed that V_3_(PET)_large particles exhibited well-defined crystalline facets, while V_3_(PET)_small particles exhibited less well-defined faceting compared to V_3_(PET)_large. Particle size analysis (Figure 2C,D) confirmed average sizes of 9 μm for V_3_(PET)_large and 311 nm for V_3_(PET)_small. The PXRD patterns of both V_3_(PET)_large and V_3_(PET)_small closely match previously reported structures, confirming that the crystallinity of the material is maintained despite size variations (Figure 2E). Nitrogen adsorption–desorption isotherms at 77 K (Figure 2F) yielded Brunauer–Emmett–Teller (BET) surface areas of 3617 m^2^/g for V_3_(PET)_large and 3474 m^2^/g for V_3_(PET)_small—values comparable to those reported for pristine V_3_(PET). To further analyze the porosity characteristics, the pore size distribution and pore volume of V_3_(PET)_large and V_3_(PET)_small were determined using the slit pore kernel model within the density functional theory (DFT) approach, based on N_2_ adsorption–desorption isotherm measurements. The DFT-calculated pore size distribution (Figures S9 and S11) indicates that both samples predominantly exhibit microporous structures, with a minor contribution from mesopores. The average pore width was determined to be approximately 1.3 nm for both particle types. The total pore volume, as determined from the N_2_ isotherm data (Figures S8 and S10), was measured to be 1.29 cm^3^/g for V_3_(PET)_large and 1.28 cm^3^/g for V_3_(PET)_small, indicating negligible differences in internal porosity despite morphological variations.

**Figure 2:**
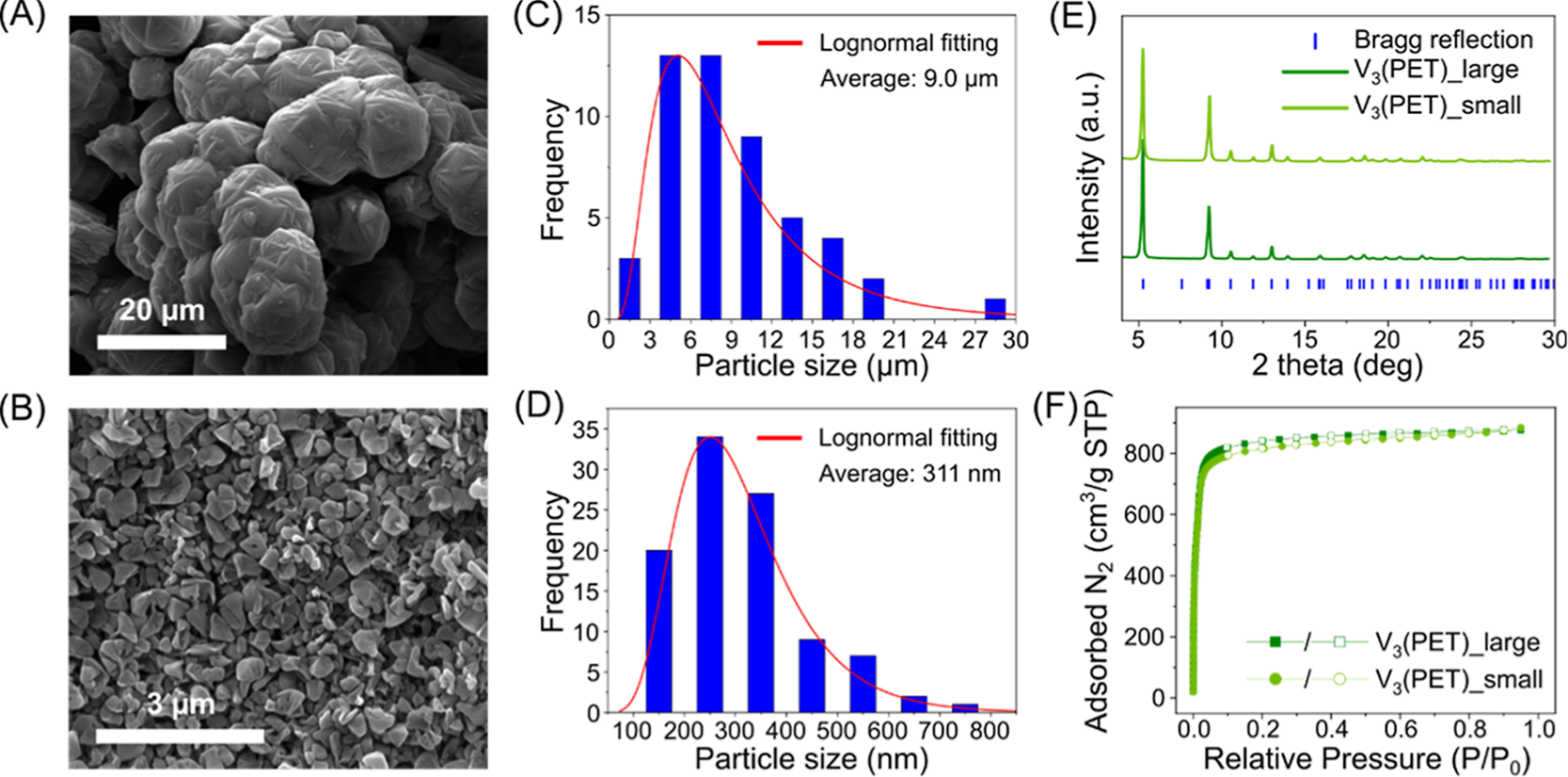
(A,B) FE-SEM images, (C,D) particle size distributions, (E) powder X-ray diffraction patterns, and (F) N_2_ adsorption–desorption isotherms (77 K) of V_3_(PET)_large and V_3_(PET)_small, respectively. (Filled symbol: adsorption, empty symbol: desorption).

### 3.3 Experimental Validation of Packing Density Enhancement

Building on the simulation-guided insights into bimodal packing, we experimentally evaluated the optimal mixing composition for maximizing the packing density of V_3_(PET) at a fixed bimodal particle size ratio. Guided by the DEM predictions (Figure 1), V_3_(PET)_large and V_3_(PET)_small were mixed in various ratios, and their tapping densities were measured with a calibrated 1 mL glass syringe. The results showed that the tapping density increased with a higher proportion of V_3_(PET)_large, reaching a maximum at a large-particle volume fraction of approximately 77.8%, which closely aligns with the optimal range predicted by the DEM simulations (∼70–80%) (Figure 3A). At this ratio, the tapping density was 0.28 g/cm^3^, corresponding to a packing fraction of 0.56—substantially higher than those of the individual components: 0.18 g/cm^3^ for V_3_(PET)_small (packing fraction = 0.35) and 0.21 g/cm^3^ for V_3_(PET)_large (packing fraction = 0.42). A photograph of the tapped V_3_(PET)_bimodal sample is shown in Figure 3B. This experimentally determined optimal ratio aligns well with both the DEM simulation results and prior literature reports on bimodal powder systems,^22^ confirming that introducing a relatively small proportion of fine particles into a coarse-particle matrix effectively fills interstitial voids and enhances overall packing. We designate this optimized bimodal mixture as V_3_(PET)_bimodal. While the experimentally achieved packing fraction (0.56) is lower than the simulated maximum (∼0.72), this difference is expected when transitioning from idealized spherical particles in DEM simulations to real MOF powders, which inherently exhibit particle shape irregularities, size polydispersity, and mild aggregation that reduce packing efficiency.^23^ Notably, the crystallographic (single-crystal) density of V_3_(PET) is 0.51 g/cm^3^, whereas the tapping bulk density is 0.28 g/cm^3^. Thus, even in the optimized case, the packing achieves only about 55% of the single-crystal density. This gap highlights the necessity of considering realistic packing behavior when evaluating material performance for volumetric storage applications, since assuming ideal packing can lead to significant overestimation of performance.

**Figure 3:**
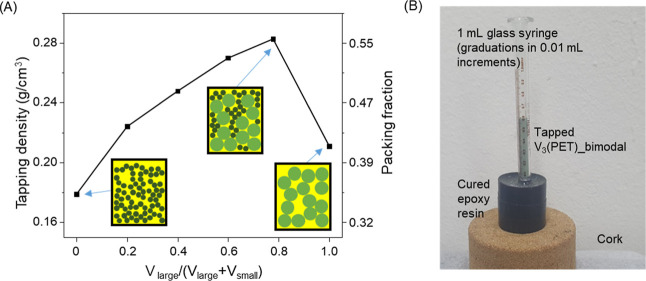
(A) Tapping density of V_3_(PET)_bimodal mixtures at various initial volume fraction of large particles. (B) Photograph of tapped V_3_(PET)_bimodal with maximum packing density in 1 mL glass syringe.

Following the determination of the optimal bimodal mixing ratio, we performed high-pressure hydrogen adsorption measurements on the three V_3_(PET) samples: unimodal large, unimodal small, and the optimized bimodal mixture. Prior to measurement, each sample was loaded into a 1.5 mL sample cell, and its packed mass was recorded to calculate the actual packing density under operational conditions. The packing densities obtained in the cell closely matched the tapping results: 0.21 g/cm^3^ for V_3_(PET)_large, 0.16 g/cm^3^ for V_3_(PET)_small, and 0.29 g/cm^3^ for V_3_(PET)_bimodal. Volumetric uptake was calculated based on the experimentally measured packing densities obtained under actual packed conditions. This consistency between the tapped and in situ packing shows that the bimodal packing strategy retains its densification advantage under practical conditions. As expected, V_3_(PET)_bimodal achieved the highest packing density of the three, experimentally validating the efficacy of bimodal particle design in reducing void volume and enhancing volumetric efficiency in MOF systems.

Furthermore, N_2_ adsorption measurements showed that the BET surface area and pore volume of V_3_(PET)_bimodal were well maintained, at 3377 m^2^/g and 1.23 cm^3^/g, respectively (Figures S12–S14), indicating that the framework integrity remained intact during packing.

### 3.4 Effect of Bimodal Packing on Hydrogen Storage Performance

With the packing density optimized, we next evaluated how this improvement translates to hydrogen adsorption performance. Prior to estimation of uptake value, helium pycnometry measurements confirmed for dead volume correction.^15^ These measurements also confirmed that the skeletal densities of V_3_(PET)_large, V_3_(PET)_small, and V_3_(PET)_bimodal are similar (1.69 g/cm^3^, 1.70 g/cm^3^, and 1.61 g/cm^3^, respectively), confirming that the formation of a bimodal particle mixture from two distinct size fractions did not alter framework-level properties such as skeletal density.

Gravimetric excess H_2_ uptake in V_3_(PET)_large, V_3_(PET)_small, and V_3_(PET)_bimodal were measured at 77, 160, and 298 K up to 100 bar (Figure S15A). At 77 K, all samples showed saturation behavior around 30–50 bar, whereas at 160 and 298 K no saturation was observed up to 100 bar. The maximum gravimetric excess uptake of V_3_(PET)_large reached approximately 5.3 wt % at around 40 bar (77 K), in good agreement with previous reports for V_3_(PET),^11^ thereby confirming the reliability of our measurements. V_3_(PET)_small exhibited a slightly higher maximum gravimetric excess uptake (∼5.7 wt % at around 40 bar and 77 K), likely due to minor differences in crystallite morphology or defect structure. In contrast, V_3_(PET)_bimodal shows slightly lower gravimetric performance (4.8 wt % at 77 K) compared to V_3_(PET)_large. Such small differences in gravimetric uptake may arise from minor variations in pore accessibility associated with particle morphology. Importantly, these variations do not translate into improved volumetric performance, primarily governed by differences in packing density. However, this gravimetric advantage of the smaller particles did not translate into improved volumetric performance, primarily due to differences in packing behavior. Figure S15B shows gravimetric total H_2_ uptake of V_3_(PET)_large, V_3_(PET)_small, and V_3_(PET)_bimodal. Similar to the excess uptake trend, V_3_(PET)_small exhibited the highest value, followed by V_3_(PET)_bimodal and then V_3_(PET)_large. At 100 bar and 77 K, the total gravimetric uptakes were approximately 9.4 wt % for V_3_(PET)_small, 8.4 wt % for V_3_(PET)_bimodal, and 8.7 wt % for V_3_(PET)_large. Compared to the excess uptake results, the differences between samples in total gravimetric uptake were relatively smaller. This is because the pore-filling gas contribution, which is added in the total uptake, moderates the disparities caused by differences in surface area and microporosity. Volumetric excess and total H_2_ uptakes estimated using the crystallographic density instead of the experimentally measured packing density (Figure S15) showed the same ordering as the gravimetric measurements: V_3_(PET)_small > V_3_(PET)_large > V_3_(PET)_bimodal. At 77 K and 100 bar, the volumetric total uptakes were approximately 48.8 g/L for V_3_(PET)_small, 43.7 g/L for V_3_(PET)_large, and 41.3 g/L for V_3_(PET)_bimodal, showing the same decreasing trend as observed in gravimetric uptake.

Despite V_3_(PET)_small exhibiting the highest intrinsic uptake, its low packing density limits its practical performance. Figure 4 illustrates how pore volume, particle volume, and interparticle voids volume determined system-level hydrogen storage. While volumetric performance is commonly discussed based on the sum of pore volume and skeletal volume, actual system-level uptake must also account for interparticle voids, which occupy a significant fraction of the total volume. These void spaces substantially reduce the effective hydrogen storage capacity in practical packed-bed systems. Therefore, evaluating hydrogen storage performance at the system level—including interparticle voids—provides a more realistic assessment than relying solely on material-level metrics, especially when designing real-world hydrogen storage systems.^24^

**Figure 4:**
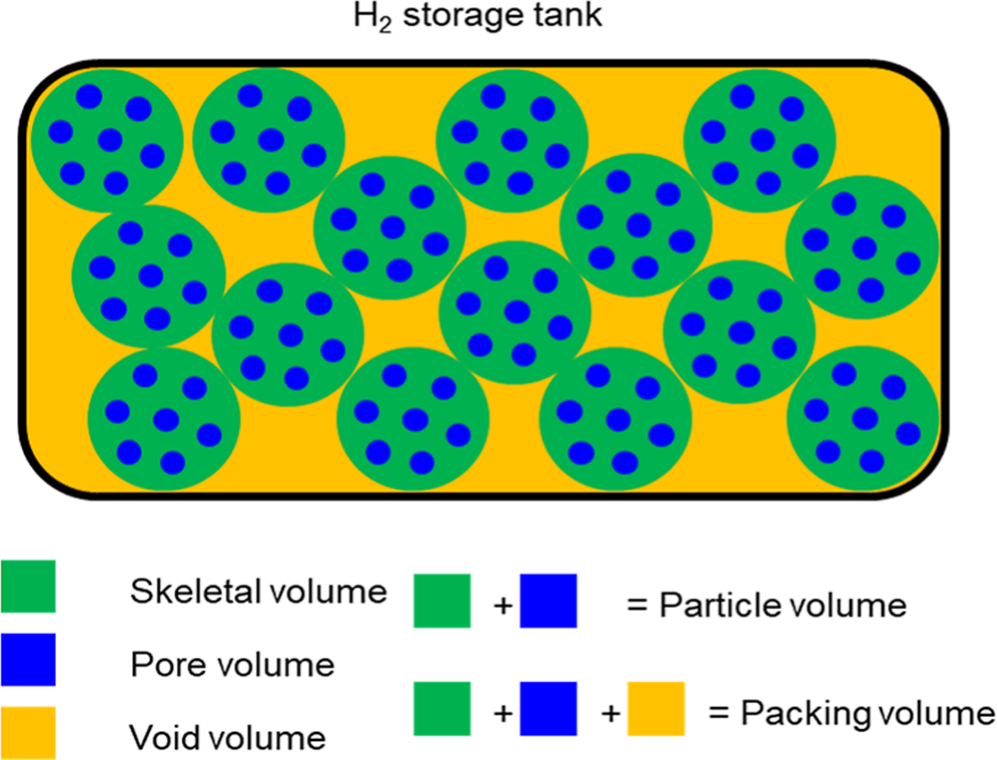
Schematic representation of system-level volume in a hydrogen tank, illustrating pore volume (blue), skeletal volume (green), and interparticle void volume (yellow).

The system-level volumetric excess and total H_2_ uptakes were calculated using the experimentally measured packing density to reflect realistic packing configurations of the MOF powders. In this calculation, the total volumetric uptake includes both the adsorbed hydrogen and the gas-phase hydrogen occupying the free volume outside the framework, including pore and interparticle void spaces, as described in the Materials and Methods section. As shown in Figure 5A, V_3_(PET)_bimodal exhibited the highest system-level volumetric excess H_2_ uptake among the three samples. At 100 bar and 77 K, V_3_(PET)_bimodal reached approximately 13.3 g/L, whereas V_3_(PET)_large and V_3_(PET)_small achieved about 10.0 g/L and 9.6 g/L, respectively. This corresponds to a volumetric excess uptake improvement of roughly 33% over V_3_(PET)_large and a 38% over V_3_(PET)_small. This significant enhancement for the bimodal sample is directly attributed to its highest packing density (0.29 g/cm^3^), which provide a greater number of adsorption sites per unit system volume. The optimized bimodal packing effectively minimizes interparticle voids while maintaining high accessibility to the pore network, leading to superior system-level volumetric storage performance compared to unimodal samples. This result highlights the importance of packing efficiency in practical hydrogen storage applications, a rationally designed particle size distribution can significantly boost system-level storage capacity, particularly in applications where volumetric capacity is a more critical metric than gravimetric capacity.^3,4,25^ With the skeletal densities of all samples being nearly identical, the observed differences in volumetric uptake can be attributed mainly to packing efficiency rather than to inherent material properties.

**Figure 5:**
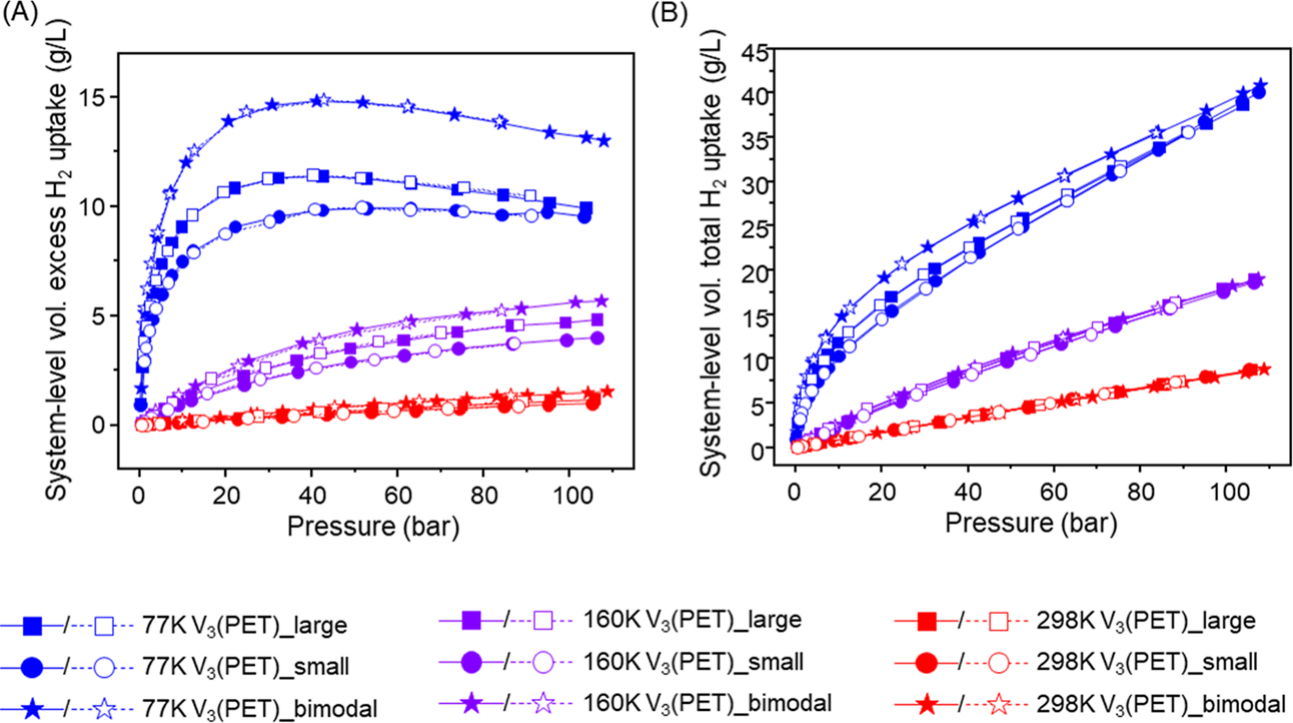
Hydrogen uptake performance of V_3_(PET)_large, V_3_(PET)_small, and V_3_(PET)_bimodal at 77, 160, and 298 K, up to 100 bar. (A) System-level volumetric excess uptake, (B) system-level volumetric total uptake (volumetric uptakes were calculated using the experimentally measured packing densities and skeletal densities).

Consistent with the trend observed for system-level volumetric excess uptake, V_3_(PET)_bimodal also exhibited the highest system-level volumetric total H_2_ uptake among the three samples (Figure 5B). At 100 bar and 77 K, the system-level volumetric total H_2_ uptake of V_3_(PET)_bimodal reached approximately 39.1 g/L, surpassing V_3_(PET)_large (37.7 g/L) and V_3_(PET)_small (38.1 g/L). Although the increase in total uptake for V_3_(PET)_bimodal appears modest (3.7% and 2.6% higher than V_3_(PET)_large and V_3_(PET)_small, respectively), this is because total uptake includes both adsorbed and non-adsorbed hydrogen within the pore volume, which is similar across all samples. In contrast, the excess uptake, which isolates the adsorption contribution by subtracting the bulk gas contribution from the total uptake, shows a significantly greater enhancement (33% and 38%, respectively), indicating more efficient utilization of volume due to improved packing. Nevertheless, the fact that V_3_(PET)_bimodal surpasses both other samples in both total and excess uptake underscores its advantage in overall hydrogen storage performance. This suggests that the bimodal system achieves a higher useable hydrogen storage density without requiring additional pore volume. This system-level efficiency is critical for practical hydrogen storage, where maximizing useable capacity per unit volume is a key design target.

Working capacity is a critical measure for evaluating the practical applicability of hydrogen storage materials. While total uptake reflects the maximum amount of hydrogen adsorbed at the charging pressure, the working capacity—defined as the deliverable amount between charging and discharging pressures—serves as a more practical metric for evaluating useable hydrogen under real-world operating conditions (Figure 6A). To assess the working performance of the V_3_(PET) series, we evaluated their working capacities under recommended representative pressure–temperature swing adsorption (PTSA) conditions.^26^ The adsorption condition was set to 77 K and 100 bar, which is widely accepted as a benchmark charging condition for cryogenic hydrogen storage. When H_2_ is desorbed at 160 K and 5 bar—conditions recommended in previous studies to maximize delivery capacity in cryogenic hydrogen storage systems—the working capacity reflects a realistic estimate of useable hydrogen under practical operating conditions.

**Figure 6:**
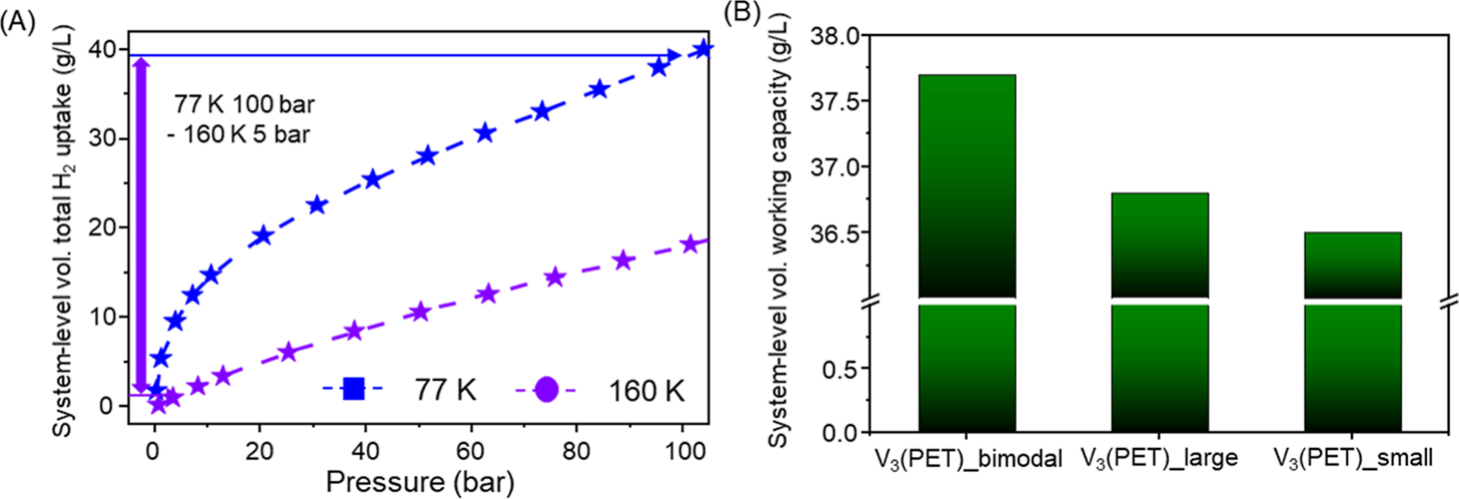
(A) Schematic illustration of working conditions of V_3_(PET)_bimodal. (B) Comparison of volumetric working capacities of V_3_(PET)_large, V_3_(PET)_small, and V_3_(PET)_bimodal under the pressure–temperature swing condition from 100 bar at 77 K to 5 bar at 160 K.

Under the PTSA condition with adsorption at 77 K and 100 bar and desorption at 160 K and 5 bar, the system-level volumetric working capacities of V_3_(PET)_bimodal, V_3_(PET)_large, and V_3_(PET)_small were 37.7, 36.8, and 36.5 g/L, respectively, with the bimodal sample showing the highest value (Figure 6B). This represents a ∼2.5% increase over V_3_(PET)_large and ∼3.3% over V_3_(PET)_small, despite the bimodal sample having a lower intrinsic gravimetric uptake. These results indicate that even modest improvements in packing efficiency can translate into gains in system-level storage capacity. This provides experimental evidence that particle packing optimization can enhance practical hydrogen storage capacity at the system level. Furthermore, this strategy has strong potential for broader applicability under various conditions, including cryogenic (77–160 K) and ambient-temperature hydrogen storage, making it a promising direction for practical implementation of adsorbent-based systems. While the absolute differences are small, the consistent advantage of the bimodal system highlights the importance of optimizing particle packing in the practical deployment of hydrogen storage materials.

## 4 Conclusion

We have demonstrated that optimizing particle packing, specifically through a bimodal particle size distribution, can substantially enhance the system-level volumetric hydrogen storage performance of MOFs. Using a V_3_(PET) as a model system, a packing fraction of 0.56 was experimentally achieved via bimodal mixing, compared to 0.42 for the unimodal form. This morphological optimization led to a 33–38% increase in volumetric excess hydrogen uptake at 77 K and enabled a working capacity of 37.7 g/L under pressure–temperature swing conditions. These results establish packing density, a parameter often underemphasized in MOF performance evaluations, as a critical and independent determinant of system-level storage efficiency, decoupled from intrinsic sorption capacity. More broadly, this work identifies particle-level packing engineering as a practical and experimentally accessible strategy for translating intrinsic materials properties into realistic storage performance. Future integration of this approach with advanced shaping or densification strategies could further narrow the gap between laboratory-scale materials metrics and the requirements of practical hydrogen storage systems. While such densification strategies can further enhance hydrogen storage performance, excessive densification may lead to increased pressure drop, and this trade-off should be considered in future system-level design.

## Supplementary Material



## Notes

Discrete element method (DEM) simulation details and quantitative packing data for bimodal MOF particle packing (Figures S1–S7 and Tables S1–S6); N_2_ adsorption and pore characterization data for V_3_(PET)_large, V_3_(PET)_small, and V_3_(PET)_bimodal (Figures S8–S14); Excess and total hydrogen uptake data for V_3_(PET)_large, V_3_(PET)_small, and V_3_(PET)_bimodal at 77, 160, and 298 K up to 100 bar (Figures S15 and S16) (PDF)

## ∇

Department of Chemistry and Chemical Biology, Cornell University, Ithaca, New York, 14853, United States
