# Supplementary material for: Enhancing Volumetric Hydrogen Storage Capacity through Bimodal Packing of MOF Particles
Source: ACS Omega. 2026 May 5;11(19):29006–14. doi: 10.1021/acsomega.6c02165 (PMC13191500; doi:10.1021/acsomega.6c02165)
Supplement: Supplementary file 1 [file ao6c02165_si_001.pdf]

Supporting Information

Enhancing Volumetric Hydrogen Storage Capacity Through Bimodal Packing of MOF Particles

Wan-Tae Kim,[1, ‡] Dae Won Kim,[2, ‡, #] Dong Yun Shin,[3] Hong-Eun An,[1, 4] Albert S. Lee,[1, 5] Jung-Hoon Lee,[3, 6] Chang Seop Hong,[2*] and Sohee Jeong[1*]

[1]Extreme Materials Research Center, Korea Institute of Science and Technology (KIST), Seoul, 02792, South Korea
[2]Department of Chemistry, Korea University, Seoul, 02841, South Korea
[3]Computational Science Research Center, Korea Institute of Science and Technology (KIST), Seoul, 02792, South Korea
[4]Department of Materials Science and Engineering, Korea University, Seoul, 02841, South Korea
[5]Convergence Research Center for Solutions to Electromagnetic Interference in Future-Mobility, Korea Institute of Science and Technology (KIST), Seoul, 02792, South Korea
[6]KU-KIST Graduate School of Converging Science and Technology, Korea University, Seoul, 02841, South Korea

Corresponding Author: cshong@korea.ac.kr (Chang Seop Hong), soheejeong@kist.re.kr (Sohee Jeong)

[‡]Wan-Tae Kim and Dae Won Kim contributed equally to this work.
[#] Present Address: Department of Chemistry and Chemical Biology, Cornell University, Ithaca, New York, 14853, United States

Section 1. discrete element method (DEM) simulations of bimodal packing behavior.

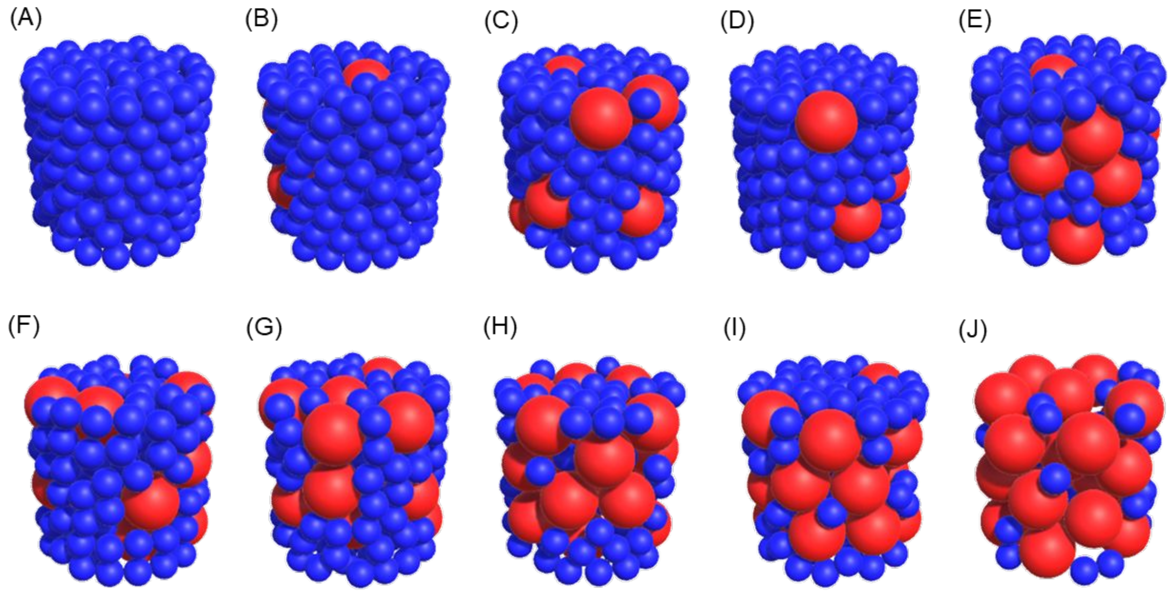

Figure S1. Representative packing structures obtained from DEM simulations at a large-to-small diameter ratio of 2:1 under varying initial volume fraction of large particles ($V_{large}/(V_{large} + V_{small})$). Red and blue spheres denote large and small particles, respectively. The structures correspond to deposition ratios of (A) 0.0, (B) 0.1, (C) 0.2, (D) 0.3, (E) 0.4, (F) 0.5, (G) 0.6, (H) 0.7, (I) 0.8, and (J) 0.9. Here, V_{large} and V_{small} denote the total volumes of the large and small particles.

Table S1. Quantitative packing data for DEM simulations at a large-to-small particle diameter ratio of 2:1 under varying $V_{large}/(V_{large} + V_{small})$.

**Initial $V_{large}/(V_{large} + V_{small})$**	0	0.1	0.2	0.3	0.4	0.5	0.6	0.7	0.8	0.9
**Cylinder volume**	785.4									
**Number of large particles**	0	4	7	6	14	14	20	23	24	25
**Volume of a single large particle**	13.83	13.83	13.83	13.83	13.83	13.83	13.83	13.83	13.83	13.83
**Total volume of large particles**	0.00	55.33	96.83	83	193.66	193.66	276.66	318.16	331.99	345.83

**Number of small particles**	262	232	209	217	150	151	113	93	79	37
**Volume of a single small particle**	1.73	1.73	1.73	1.73	1.73	1.73	1.73	1.73	1.73	1.73
**Total volume of small particles**	453.03	401.16	361.39	375.22	259.37	261.1	195.39	160.81	136.6	63.98
**Total packed particle volume**	453.03	456.49	458.22	458.22	453.03	454.76	472.05	478.97	468.6	409.8
**Packing fraction**	0.58	0.58	0.58	0.58	0.58	0.58	0.6	0.61	0.6	0.52
**Final $V_{large}/(V_{large} + V_{small})$**	0	0.12	0.21	0.18	0.43	0.43	0.59	0.66	0.71	0.84

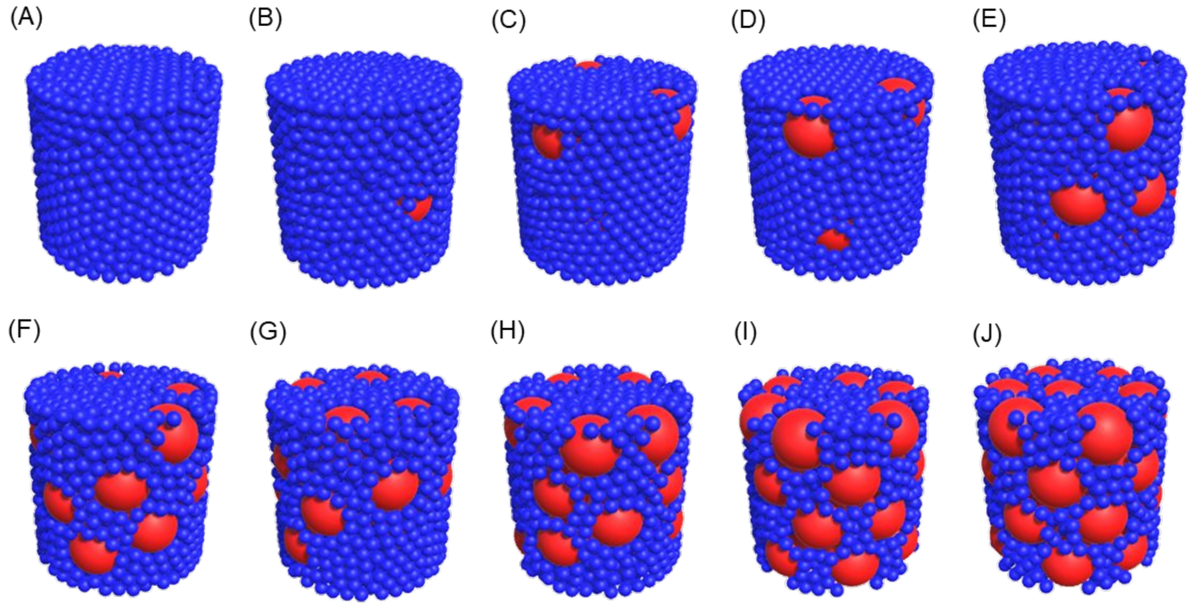

Figure S2. Representative packing structures obtained from DEM simulations at a large-to-small diameter ratio of 4:1 under varying initial volume fraction of large particles ($V_{large}/(V_{large} + V_{small})$). Red and blue spheres denote large and small particles, respectively. The structures correspond to deposition ratios of (A) 0.0, (B) 0.1, (C) 0.2, (D) 0.3, (E) 0.4, (F) 0.5, (G) 0.6, (H) 0.7, (I) 0.8, and (J) 0.9.

Table S2. Quantitative packing data for DEM simulations at a large-to-small particle diameter ratio of 4:1 under varying $V_{large}/(V_{large} + V_{small})$.

**Initial $V_{large}/(V_{large} + V_{small})$**	0	0.1	0.2	0.3	0.4	0.5	0.6	0.7	0.8	0.9
**Cylinder volume**	785.4									
**Number of large particles**	0	2	10	9	16	19	20	24	27	27
**Volume of a single large particle**	13.83	13.83	13.83	13.83	13.83	13.83	13.83	13.83	13.83	13.83
**Total volume of large particles**	0	27.67	138.33	124.5	221.33	262.83	276.66	331.99	373.49	373.49
**Number of small particles**	2211	2005	1629	1671	1281	1046	1008	846	675	609
**Volume of a single small particle**	0.22	0.22	0.22	0.22	0.22	0.22	0.22	0.22	0.22	0.22
**Total volume of small particles**	477.89	433.36	352.09	361.17	276.88	226.08	217.87	182.86	145.9	131.63
**Total packed particle volume**	477.89	461.03	490.43	485.67	498.21	488.91	494.53	514.85	519.39	505.12
**Packing fraction**	0.61	0.59	0.62	0.62	0.63	0.62	0.63	0.66	0.66	0.64
**Final $V_{large}/(V_{large} + V_{small})$**	0	0.06	0.28	0.26	0.44	0.54	0.56	0.64	0.72	0.74

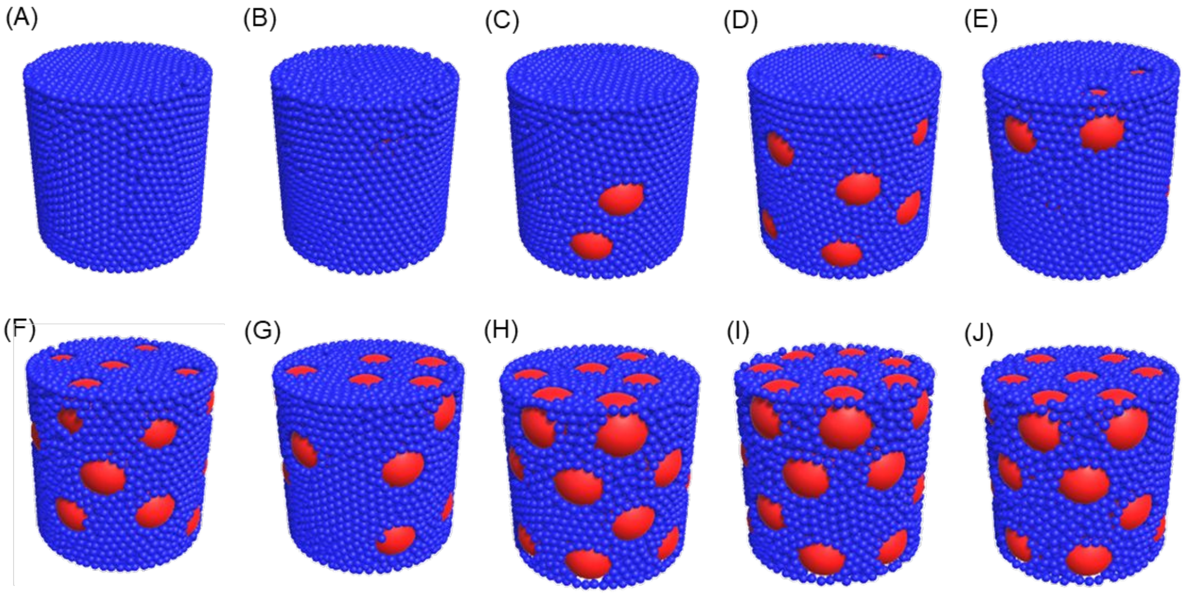

Figure S3. Representative packing structures obtained from DEM simulations at a large-to-small diameter ratio of 6:1 under varying initial volume fraction of large particles ($V_{large}/(V_{large} + V_{small})$). Red and blue spheres denote large and small particles, respectively. The structures correspond to deposition ratios of (A) 0.0, (B) 0.1, (C) 0.2, (D) 0.3, (E) 0.4, (F) 0.5, (G) 0.6, (H) 0.7, (I) 0.8, and (J) 0.9.

Table S3. Quantitative packing data for DEM simulations at a large-to-small particle diameter ratio of 6:1 under varying $V_{large}/(V_{large} + V_{small})$.

**Initial $V_{large}/(V_{large} + V_{small})$**	0	0.1	0.2	0.3	0.4	0.5	0.6	0.7	0.8	0.9
**Cylinder volume**	785.4									
**Number of large particles**	0	2	10	13	17	23	24	27	27	27
**Volume of a single large particle**	13.83	13.83	13.83	13.83	13.83	13.83	13.83	13.83	13.83	13.83
**Total volume of large particles**	0	27.67	138.33	179.83	235.16	318.16	331.99	373.49	373.49	373.49
**Number of small particles**	6759	6239	4886	4360	3609	3335	3068	2470	2468	2029

**Volume of a single small particle**	0.06	0.06	0.06	0.06	0.06	0.06	0.06	0.06	0.06	0.06
**Total volume of small particles**	432.86	399.56	312.91	279.22	231.13	213.58	196.48	158.18	158.06	129.94
**Total packed particle volume**	432.86	427.22	451.24	459.05	466.29	531.74	528.47	531.68	531.55	503.43
**Packing fraction**	0.55	0.54	0.57	0.58	0.59	0.68	0.67	0.68	0.68	0.64
**Final $V_{large}/(V_{large} + V_{small})$**	0	0.06	0.31	0.39	0.5	0.6	0.63	0.7	0.7	0.74

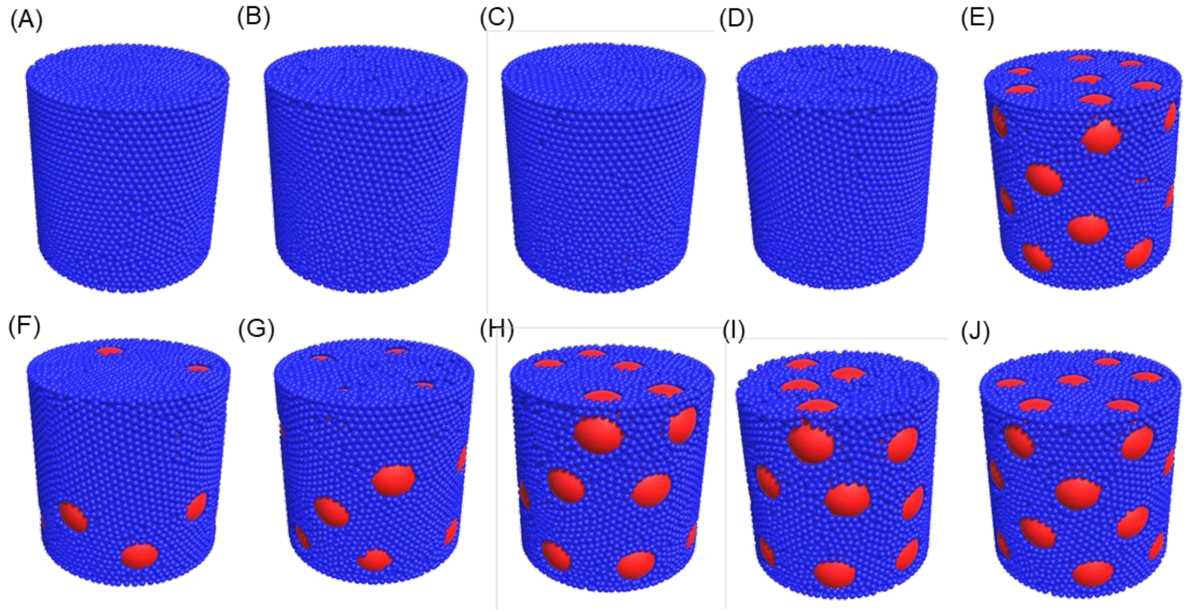

Figure S4. Representative packing structures obtained from DEM simulations at a large-to-small diameter ratio of 8:1 under varying initial volume fraction of large particles ($V_{large}/(V_{large} + V_{small})$). Red and blue spheres denote large and small particles, respectively. The structures correspond to deposition ratios of (A) 0.0, (B) 0.1, (C) 0.2, (D) 0.3, (E) 0.4, (F) 0.5, (G) 0.6, (H) 0.7, (I) 0.8, and (J) 0.9.

Table S4. Quantitative packing data for DEM simulations at a large-to-small particle diameter ratio of 8:1 under varying $V_{large}/(V_{large} + V_{small})$.

**Initial $V_{large}/(V_{large} + V_{small})$**	0	0.1	0.2	0.3	0.4	0.5	0.6	0.7	0.8	0.9
**Cylinder volume**	785.4									
**Number of large particles**	0	4	8	11	17	25	27	27	28	28
**Volume of a single large particle**	13.83	13.83	13.83	13.83	13.83	13.83	13.83	13.83	13.83	13.83
**Total volume of large particles**	0	55.33	110.66	152.16	235.16	345.83	373.49	373.49	387.33	387.33
**Number of small particles**	17188	15527	13923	12526	3609	7432	6595	6505	5776	5099
**Volume of a single small particle**	0.03	0.03	0.03	0.03	0.06	0.03	0.03	0.03	0.03	0.03
**Total volume of small particles**	446.05	402.95	361.32	325.07	231.13	192.87	171.15	168.81	149.89	132.33
**Total packed particle volume**	446.05	458.28	471.98	477.23	466.29	538.7	544.64	542.31	537.22	519.65
**Packing fraction**	0.57	0.58	0.6	0.61	0.59	0.69	0.69	0.69	0.68	0.66
**Final $V_{large}/(V_{large} + V_{small})$**	0	0.12	0.23	0.32	0.5	0.64	0.69	0.69	0.72	0.75

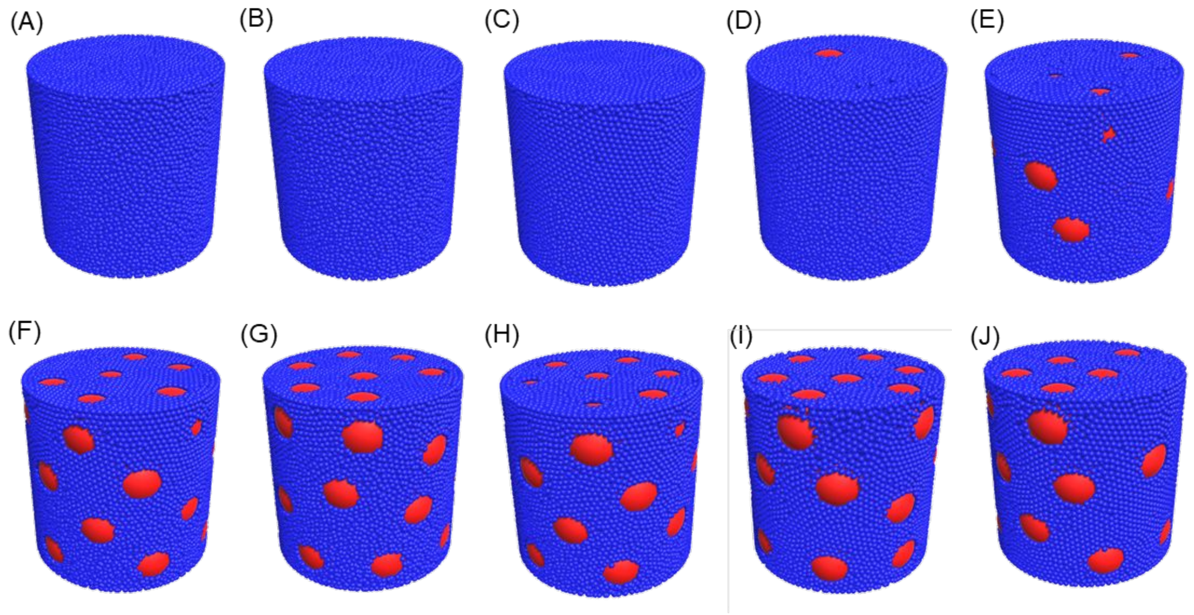

Figure S5. Representative packing structures obtained from DEM simulations at a large-to-small diameter ratio of 10:1 under varying initial volume fraction of large particles ($V_{large}/(V_{large} + V_{small})$). Red and blue spheres denote large and small particles, respectively. The structures correspond to deposition ratios of (A) 0.0, (B) 0.1, (C) 0.2, (D) 0.3, (E) 0.4, (F) 0.5, (G) 0.6, (H) 0.7, (I) 0.8, and (J) 0.9.

Table S5. Quantitative packing data for DEM simulations at a large-to-small particle diameter ratio of 10:1 under varying $V_{large}/(V_{large} + V_{small})$.

**Initial $V_{large}/(V_{large} + V_{small})$**	0	0.1	0.2	0.3	0.4	0.5	0.6	0.7	0.8	0.9
**Cylinder volume**	785.4									
**Number of large particles**	0	5	9	12	18	26	27	28	28	28
**Volume of a single large particle**	13.83	13.83	13.83	13.83	13.83	13.83	13.83	13.83	13.83	13.83
**Total volume of large particles**	0	69.17	124.5	166	249	359.66	373.49	387.33	387.33	387.33
**Number of small particles**	32559	28582	25264	22559	18471	14700	13523	13050	12289	10380

**Volume of a single small particle**	0.01	0.01	0.01	0.01	0.01	0.01	0.01	0.01	0.01	0.01
**Total volume of small particles**	450.39	395.38	349.48	312.06	255.51	203.35	187.06	180.52	169.99	143.59
**Total packed particle volume**	450.39	464.54	473.98	478.06	504.51	563.01	560.56	567.85	557.32	530.91
**Packing fraction**	0.57	0.59	0.6	0.61	0.64	0.72	0.71	0.72	0.71	0.68
**Final $V_{large}/(V_{large} + V_{small})$**	0	0.15	0.26	0.35	0.49	0.64	0.67	0.68	0.69	0.73

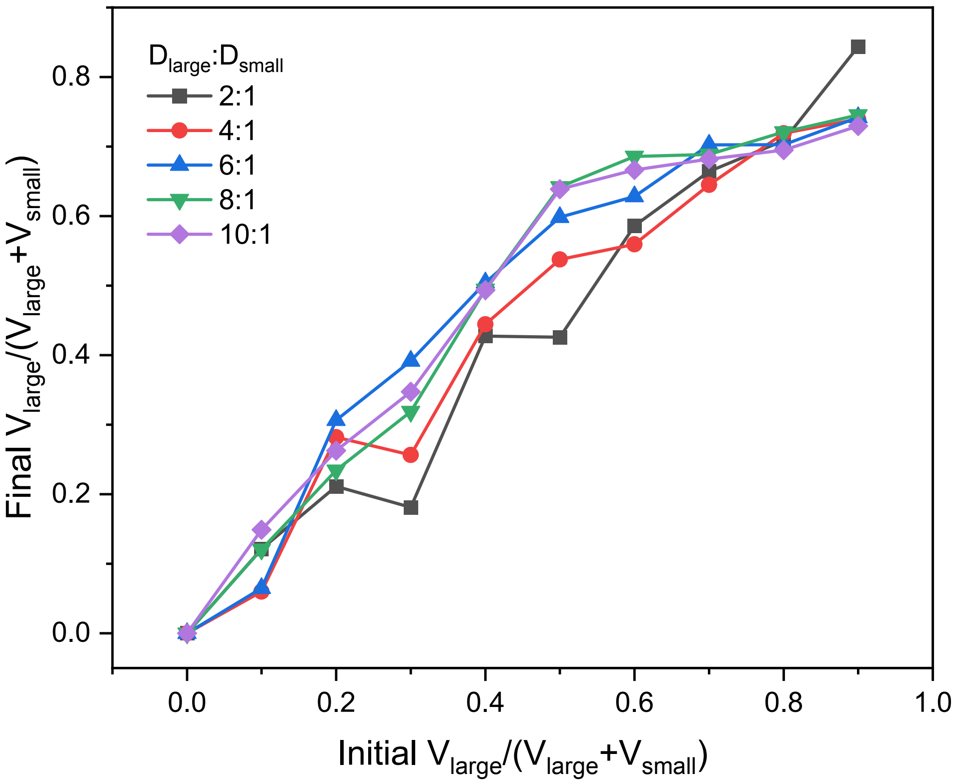

Figure S6. Final volume fraction of large particles, V_{large} / (V_{large} + V_{small}), as a function of the initial volume fraction for different particle size ratios obtained from DEM simulations.

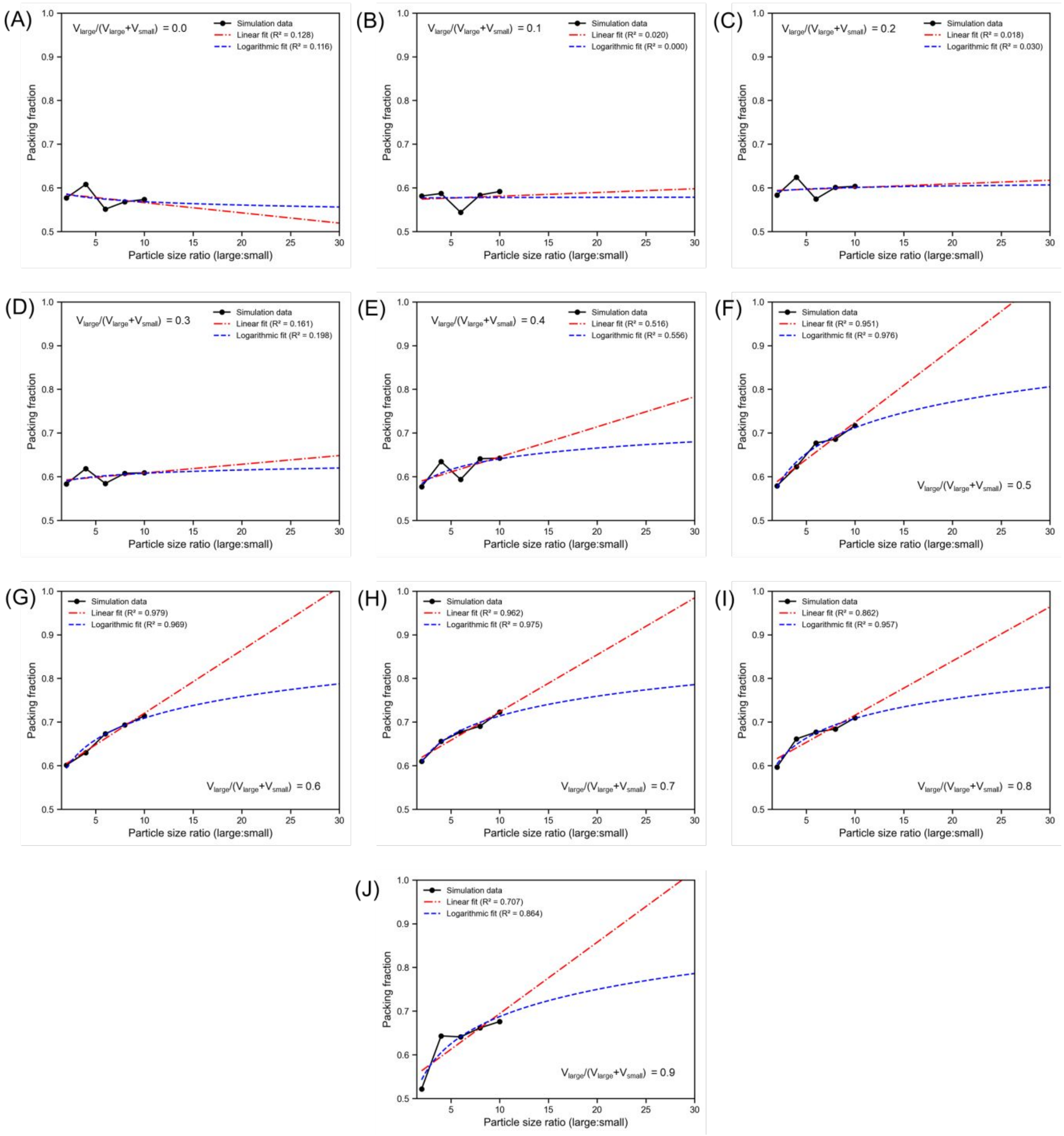

Figure S7. Packing fraction as a function of particle size ratio (D_{large}:D_{small}) at different initial volume fractions of large particles (V_{large} / (V_{large} + V_{small})). Simulation data are shown as black symbols, with linear (red dash–dot lines) and logarithmic (blue dashed lines) fitting results. The data were fitted using linear (y = a + bx) and logarithmic (y = a + b ln(x)) models.

Table S6. Packing fraction values obtained from linear and logarithmic fitting of the simulation as a function of particle size ratio (D_{large}:D_{small}) at different initial volume fractions of large particles (V_{large} / (V_{large} + V_{small})).

V_{large} / (V_{large} + V_{small})		0	0.1	0.2	0.3	0.4	0.5	0.6	0.7	0.8	0.9
Packing fraction	Linear fit	0.519	0.598	0.617	0.648	0.783	1.063	1.009	0.985	0.964	1.021
	Logarithmic fit	0.556	0.578	0.607	0.62	0.68	0.806	0.787	0.786	0.78	0.786

Section 2. N_2 adsorption and pore characterization of V_3(PET)_large, V_3(PET)_small, and V_3(PET)_bimodal.

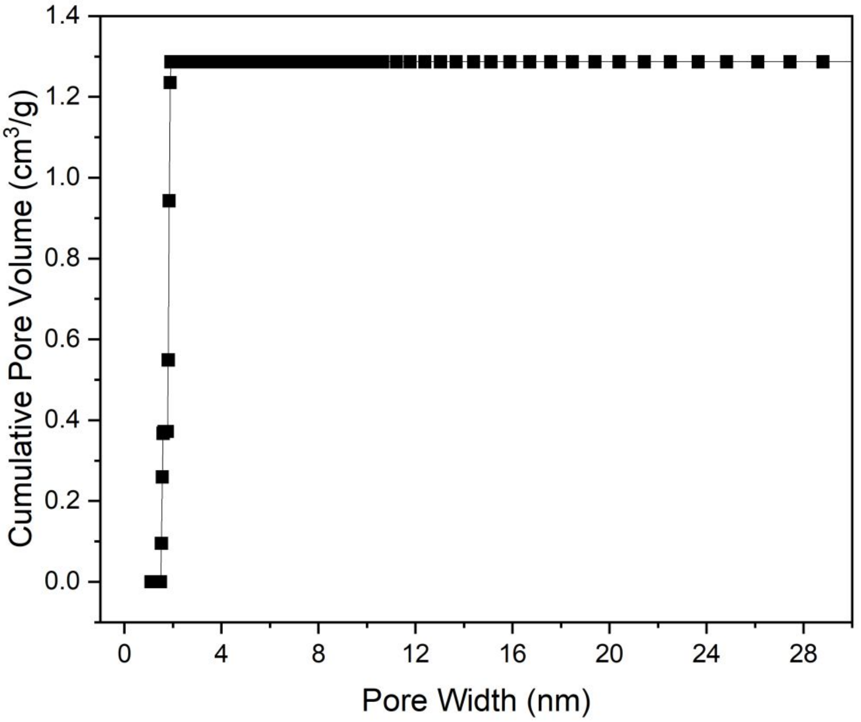

Figure S8. Cumulative pore volume of V_3(PET)_large.

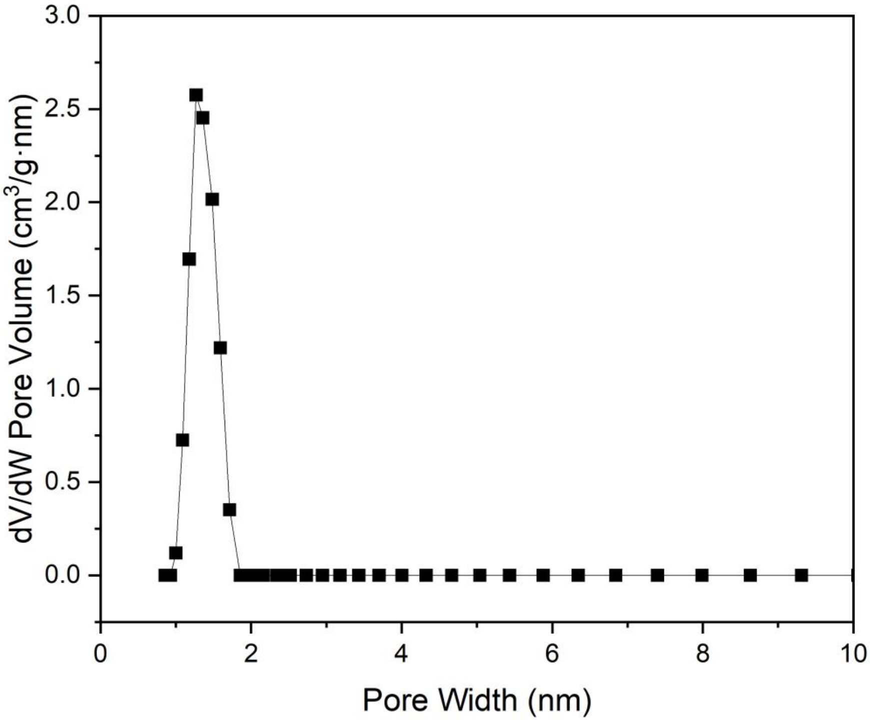

Figure S9. Pore size distribution of V_3(PET)_large.

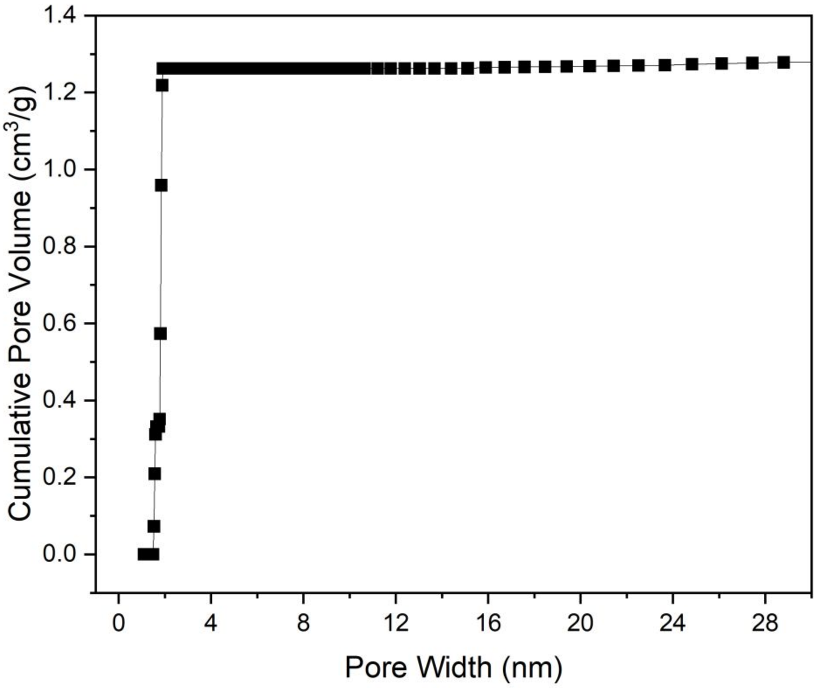

Figure S10. Cumulative pore volume of V_3(PET)_small.

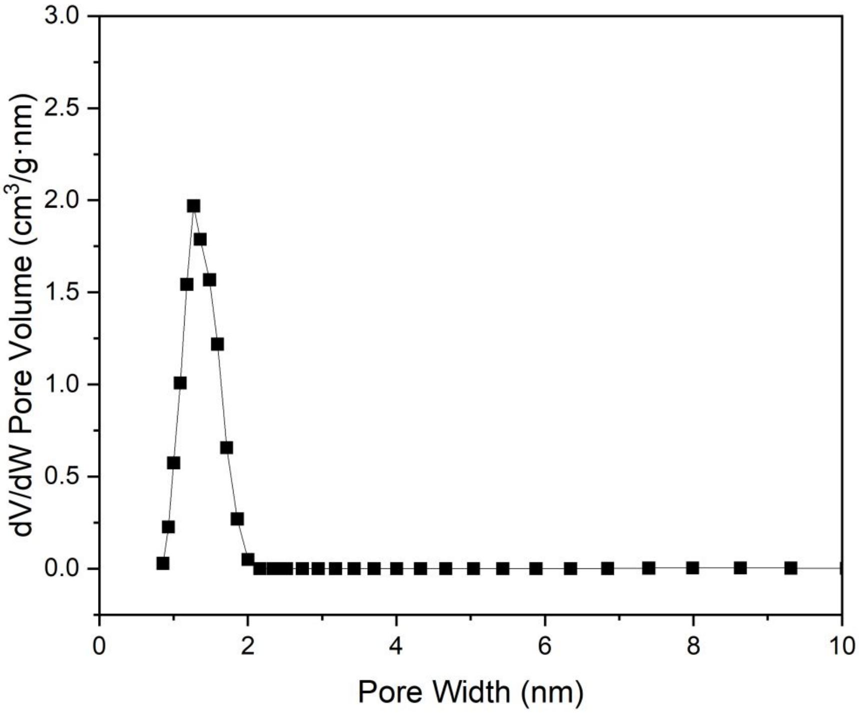

Figure S11. Pore size distribution of V_3(PET)_small.

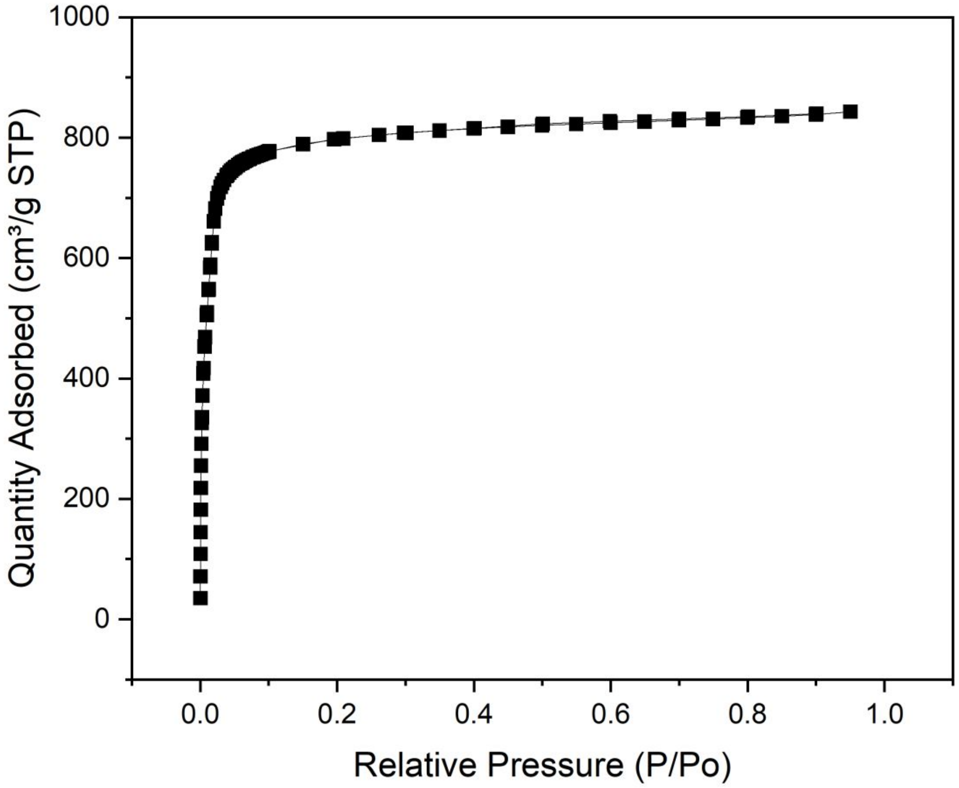

Figure S12. N_2 isotherm of V_3(PET)_bimodal at 77K.

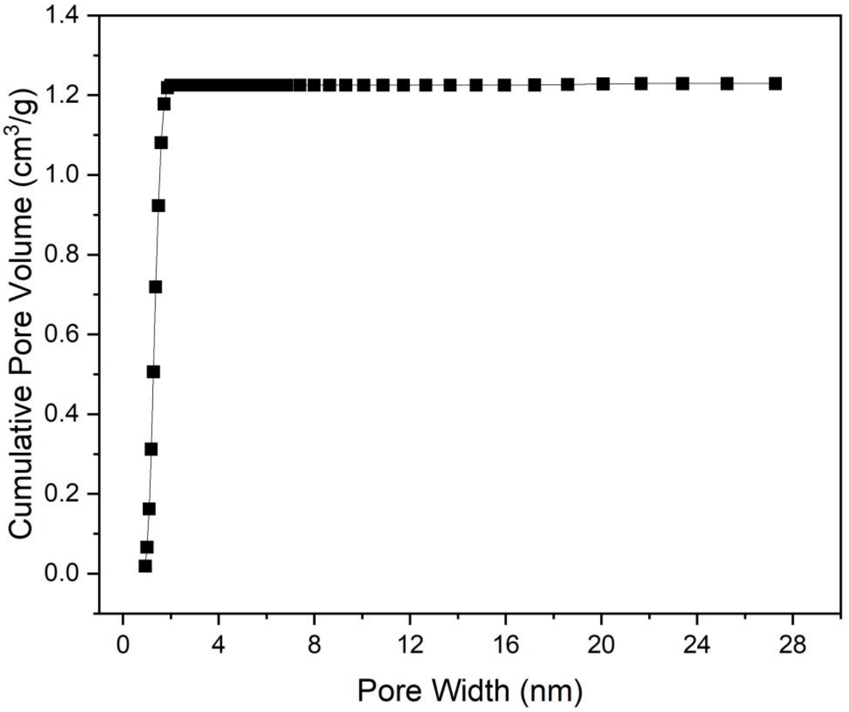

Figure S13. Cumulative pore volume of V_3(PET)_bimodal.

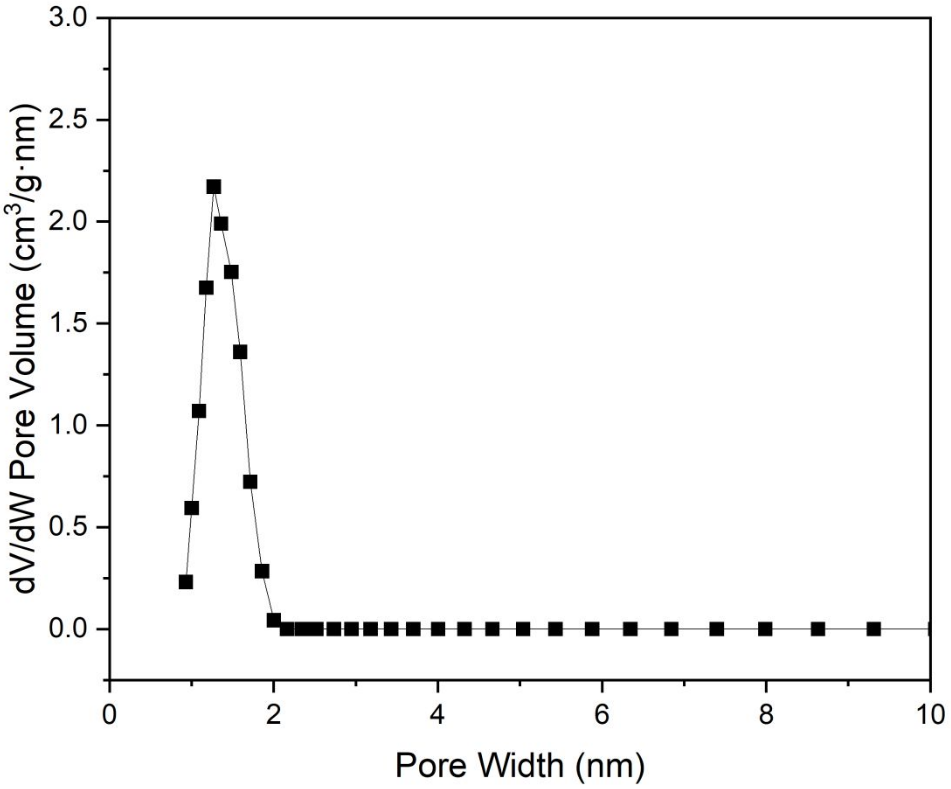

Figure S14. Pore size distribution of V_3(PET)_bimodal.

Section 3. excess and total hydrogen uptake of V_3(PET)

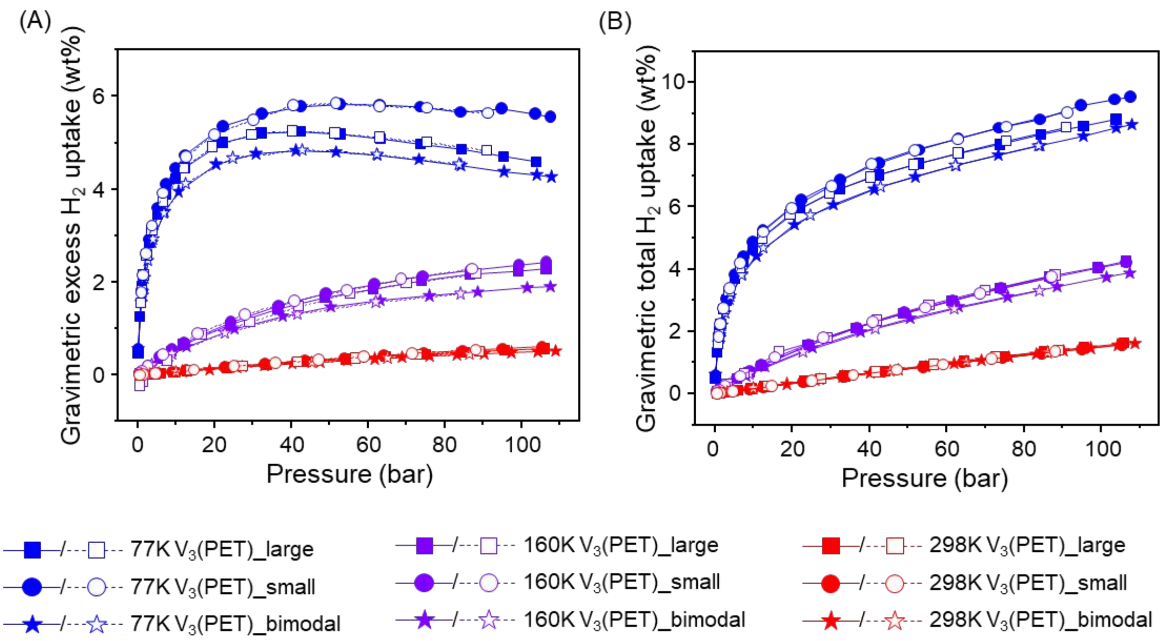

Figure S15. Hydrogen uptake performance of V_3(PET)_large, V_3(PET)_small, and V_3(PET)_bimodal at 77, 160, and 298 K, up to 100 bar. (A) Gravimetric excess uptake, (B) gravimetric total uptake.

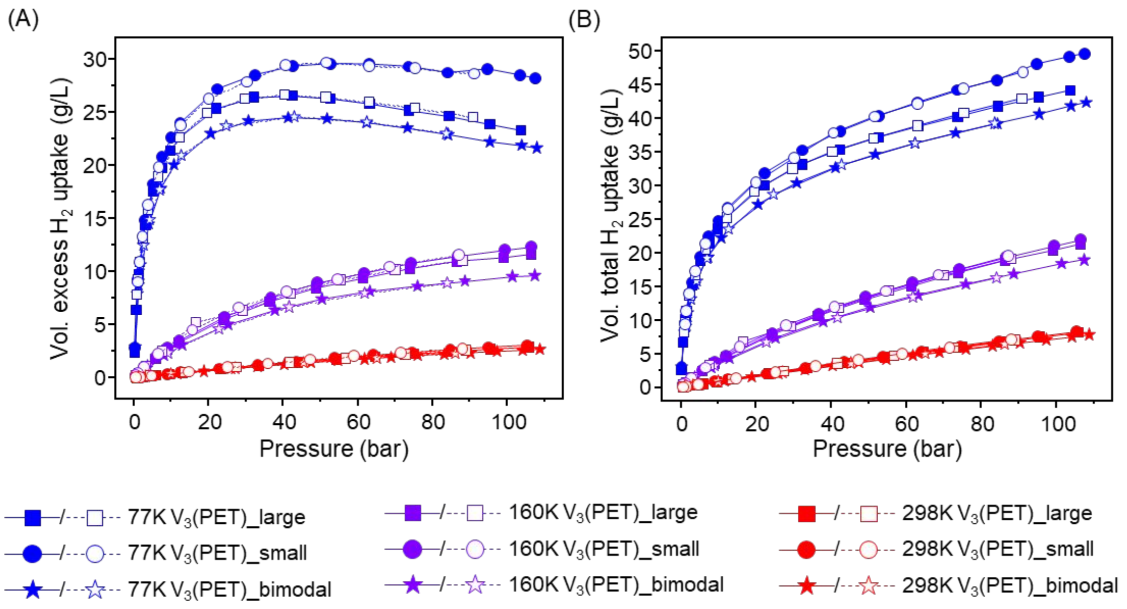

Figure S16. Hydrogen uptake performance of V_3(PET)_large, V_3(PET)_small, and V_3(PET)_bimodal at 77, 160, and 298 K, up to 100 bar. (A) Volumetric excess uptake, (B) volumetric total uptake.
